# Local Variations in Spatial Synchrony of Influenza Epidemics

**DOI:** 10.1371/journal.pone.0043528

**Published:** 2012-08-16

**Authors:** James H. Stark, Derek A. T. Cummings, Bard Ermentrout, Stephen Ostroff, Ravi Sharma, Samuel Stebbins, Donald S. Burke, Stephen R. Wisniewski

**Affiliations:** 1 Division of Epidemiology, New York City Department of Health and Mental Hygiene, New York, New York, United States of America; 2 Department of Epidemiology, Bloomberg School of Public Health, Johns Hopkins University, Baltimore, Maryland, United States of America; 3 Department of Mathematics, School of Arts and Sciences, University of Pittsburgh, Pittsburgh, Pennsylvania, United States of America; 4 Bureau of Epidemiology, Pennsylvania Department of Health, Harrisburg, Pennsylvania, United States of America; 5 Department of Behavioral and Community Health Sciences, Graduate School of Public Health, University of Pittsburgh, Pittsburgh, Pennsylvania, United States of America; 6 Department of Epidemiology, Graduate School of Public Health, University of Pittsburgh, Pittsburgh, Pennsylvania, United States of America; National University of Singapore, Singapore

## Abstract

**Background:**

Understanding the mechanism of influenza spread across multiple geographic scales is not complete. While the mechanism of dissemination across regions and states of the United States has been described, understanding the determinants of dissemination between counties has not been elucidated. The paucity of high resolution spatial-temporal influenza incidence data to evaluate disease structure is often not available.

**Methodology and Findings:**

We report on the underlying relationship between the spread of influenza and human movement between counties of one state. Significant synchrony in the timing of epidemics exists across the entire state and decay with distance (regional correlation = 62%). Synchrony as a function of population size display evidence of hierarchical spread with more synchronized epidemics occurring among the most populated counties. A gravity model describing movement between two populations is a stronger predictor of influenza spread than adult movement to and from workplaces suggesting that non-routine and leisure travel drive local epidemics.

**Conclusions:**

These findings highlight the complex nature of influenza spread across multiple geographic scales.

## Introduction

Despite the regularity of influenza epidemics, understanding the nature of influenza spread remains unclear. Inferences reflecting the spatiotemporal patterns of disease spread have been advanced in recent years through availability of detailed spatial-temporal data and the application of synchrony and time-frequency decomposition methods [1], [2]. Evidence of spatial synchrony and traveling waves have been reported in infectious diseases such as measles and dengue resulting in novel insights into urban and rural infection hierarchies and the impact of spatial heterogeneities of the host population of incidence waves [1], [3], [4]. These approaches have been extended to influenza which has observed population density, human movement, and antigenic dominance as key determinants of influenza spread at the country scale [5], [6], [7], [8], [9].

The current understanding of the intrinsic properties of influenza epidemics is limited by the geographic scales used to evaluate the data. Often the spatial scale of analysis is the continent or country [6], [8], [10]. Analyses conducted at larger spatial scales may potentially conceal local trends in disease structure. High resolution spatial-temporal infection data is often not available. As a result, there are few opportunities to validate findings at large spatial scales with finer spatial scale observations. The mechanism of influenza spread is one such example. Brownstein et al. showed the importance of air travel in the dissemination of influenza cases across census regions in the United States [9]. Viboud et al. used state-specific mortality data to demonstrate the relative importance of workflows compared to distance and other movement metrics in capturing the spatial synchrony of influenza mortality in the United States [6]. While these finding are relevant to understanding the spread of influenza within the United States, confirmation of these results using more spatially refined incidence data would test the consistency of these relationships across a broad geographic spectrum.

Gravity models have been used to explain spatial dynamics of epidemics [6], [11], [12], [13]. They were developed in transportation theory to model the flow of travelers across a landscape [14]. The gravity model describes the magnitude of travel between two locations as a function of the population sizes in the two locations and the distance between those locations. Because a gravity model estimates a general pattern of movement without preconditions on type or geographic features of the location, evaluation of a gravity-model may provide insight into local interactions not captured by well-defined mechanisms of travel.

In this report, laboratory confirmed influenza cases from Pennsylvania, United States are used to compare county-specific incidence patterns. As the sixth most populated state in the United States, Pennsylvania is divided among 67 counties, of which two counties, Allegheny and Philadelphia, account for greater than 22% of the state’s population. The state is trifurcated by two major interstate highways with limited transportation networks in the northern counties and has international airports on opposite ends of the state. With extreme segmentation in the population structure and a divisive transportation network, Pennsylvania is a unique locale to assess the predictors of influenza spread at a local level.

This is the first report to evaluate the underlying relationship of disease spread and human movement using county-specific influenza cases. Estimates of spatial synchrony are evaluated using correlation coefficients and the Mantel statistic to determine whether synchrony is associated with large numbers of adult workflows or gravity-like estimates of interaction. Understanding the mechanism of spread at a fine spatial scale would provide an improved level of understanding not previously available for local, county and city public health officials to implement surveillance and response activities.

## Methods

### Data

**Figure 1:**
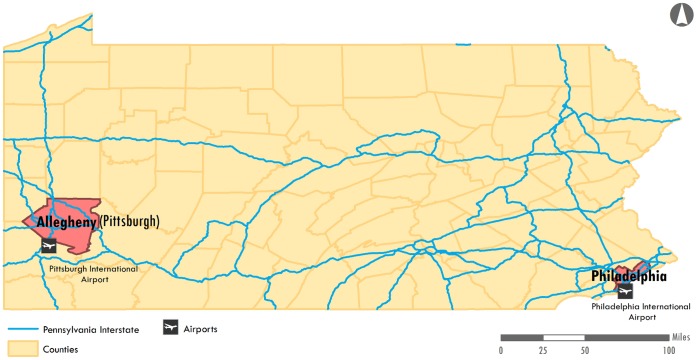
Map of Pennsylvania, US detailing the county boundaries, urban areas, and transportation networks. Pittsburgh in Allegheny County is highlighted in the West and Philadelphia is highlighted in the South East. Both urban areas have international airports and are connected by a major interstate highway.

Weekly estimates of reported influenza cases from 2003–2009 were provided by the Pennsylvania Department of Health. Briefly, the Pennsylvania National Electronic Disease Surveillance System (PA-NEDSS) is a computer application used to conduct surveillance of reportable diseases including influenza. Case reports are routinely collected by providers and laboratories and are transmitted electronically to the PA-NEDSS system. The surveillance system defines each influenza season to begin in the 40^th^ week of the calendar year through the last week of April of the following year. Influenza data occurring during this entire time period were used for this analysis. Cases were aggregated by week and to one of 67 Pennsylvania counties, respectively. The analysis for this study used 186 weeks of surveillance data accumulated over 6 seasons (31 weeks/year). The total number of reported influenza cases during the study period (2003–2009) was 57,598. A map illustrating the features of Pennsylvania has been included as Figure 1. The US census provided annual population estimates to calculate seasonal incidence for each county [15]. Rates of human work flux data between counties for the year 2000 was obtained from the US Census [16]. The workflow data describes in which county people work and in which county they reside; thus approximations of flow between counties could be calculated.

### Synchrony and Mantel Correlation Analysis

Spatial synchrony provides an estimate of the correlation of an epidemic time series across a geographic region [1], [17]. For this analysis, spatial synchrony was measured as the Spearman rank correlation of the pairwise comparisons of weekly cases for each county over the entire study period. Algorithms for the spatial correlation function estimating the relationship between synchrony and Euclidean distance were obtained from the NCF library for R, specifically the non-parametric covariance function [18], [19].

Often linked with ecological and environmental analyses, Mantel tests are used to describe the distribution of species and their association with environmental and geographic attributes. Typically, the inherent autocorrelation of such predictors constrains traditional analytic approaches. However, the Mantel test is a regression which characterizes each variable as a dissimilarity or distance matrix describing the pair-wise relationship between locations [20]. For example, a predictor variable describes the dissimilarity of population values at locations i and j. For this analysis, the question of interest is whether locations with similar influenza epidemics can be explained by similarities in other identifiable characterizes between those same locations. Thus, Mantel tests were used to compare the matrix of pair-wise Spearman correlations of influenza time series to matrices describing pair wise county to county human movement, geographic distance, and population size [21].

The Mantel statistics estimated the correlation of the comparative elements between two 67×67 matrices. For every pair of counties, a Spearman correlation was generated between each 186 week time series. As a result, the influenza matrix consisted of pair-wise correlations for 67 county pairs; a total of 4489 Spearman correlations. A separate Mantel test was conducted between the influenza matrix and each predictor variable. A workflow matrix was composed of the number of individuals who reported commuting from county i to county j in the US Census dataset by summing the movement to and from each county resulting in a symmetric 67×67 matrix. Distances between counties were represented by a Euclidian distance matrix based on the geographic centroid for each county. The population matrix consisting of the product of counties i and j was also tested. Partial Mantel’s test, a technique theoretically similar to a multiple regression, was used to measure the association of two matrices in the presence of a third matrix. In essence, the Partial Mantel’s test estimated the contribution of a second independent variable in the presence of the first independent variable.

Two Pennsylvania counties may have limited movement between one another but may engage in substantial workflow contact through a third non-Pennsylvania county; thus having an indirect effect on the epidemic synchrony. In order to explore whether this workflow might explain the pattern of correlations of influenza observed in Pennsylvania, an additional workflow matrix capturing these second-order movements (inter-state) for counties in border states was created and included in the Mantel tests. This matrix incorporated workflows to and from 302 counties from the six states bordering Pennsylvania (Delaware, Maryland, Ohio, New Jersey, New York, and West Virginia).

We estimated the exponents of a gravity model that maximized the Mantel correlation of the gravity model with the disease spread or workflow matrices using Nelder-Mead optimization. This optimization procedure searches for the local minimum of a function of interdependent variables (population and distance) through continually refining the vertices of a multi-dimensional trangle (simplex) derived from a set of starter values [22].
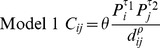



A gravity model for either workflows or disease spread (C_ij_) was parameterized by the population of counties i and j (P_i_, P_j_) and the distance between the two counties (D_ij_) (Model 1). The exponents τ1, τ2, and ρ, estimated by the model, quantify the attraction of the receiving and generating counties by population size and the distance between two counties. Theta, θ, is the proportionality constant.

The Mantel test compared the pair-wise Spearman correlations of influenza time series and the gravity matrix. An additional Mantel statistic measured the association of influenza time series and a matrix based on a gravity model that used Pennsylvania population and distance parameters and the exponents from an established gravity model developed from national influenza mortality data [6]. The purpose of this matrix was to determine whether the gravity model observed in national influenza data could describe the movement patterns seen at a finer spatial scale.

### Sampling

Small case counts from counties with a small population may have resulted in increased sampling variability and poor correlation with other counties. Even with uniform reporting efficiencies in each county, we expected small counties to report more weeks with zero cases which may have led to greater variability. Thus, it would have been difficult to differentiate the effect of population size and reporting error on disease spread. To address these concerns, a sampling method adapted from Grassly et al. was employed to test if the differences in influenza epidemics could be attributed to reporting error [23].

To appropriately evaluate the effect of reporting error, we constructed a time series with additional sampling error for the largest populated counties using the binomial distribution. For the 30 counties with the largest populations, the reported incidence rates at each time point for each of the 30 counties was resampled 1,000 times from a binomial distribution with a sample size equal to the remaining 37 counties (randomly sampled with replacement). The selection of 30 counties for the resampling was based on a natural break in the distribution of population sizes. This resulted in a new time series for each of the 30 larger populated counties as if they had sampling error equivalent to the 37 smaller populated counties. Next, 1000 pair-wise Spearman correlation matrices were created from the binomial-generated time series. The average correlation was calculated from each new correlation matrix and they were ranked to obtain the 25^th^ and 975^th^ values, in essence a confidence interval. This distribution was compared with the mean correlation of the observed correlation matrix for the 37 smaller populated counties. A statistically significant difference in the correlation between large populated and small populated counties will result if the distribution of binomial sampled correlations excludes the mean correlation of the smaller populated counties. Thus, a statistically significant result is not likely to reflect differences in sampling error and provide further confidence in the synchrony and correlation analysis.

Institutional review board approval was obtained from the Pennsylvania Department of Health and the University of Pittsburgh.

## Results

**Figure 2:**
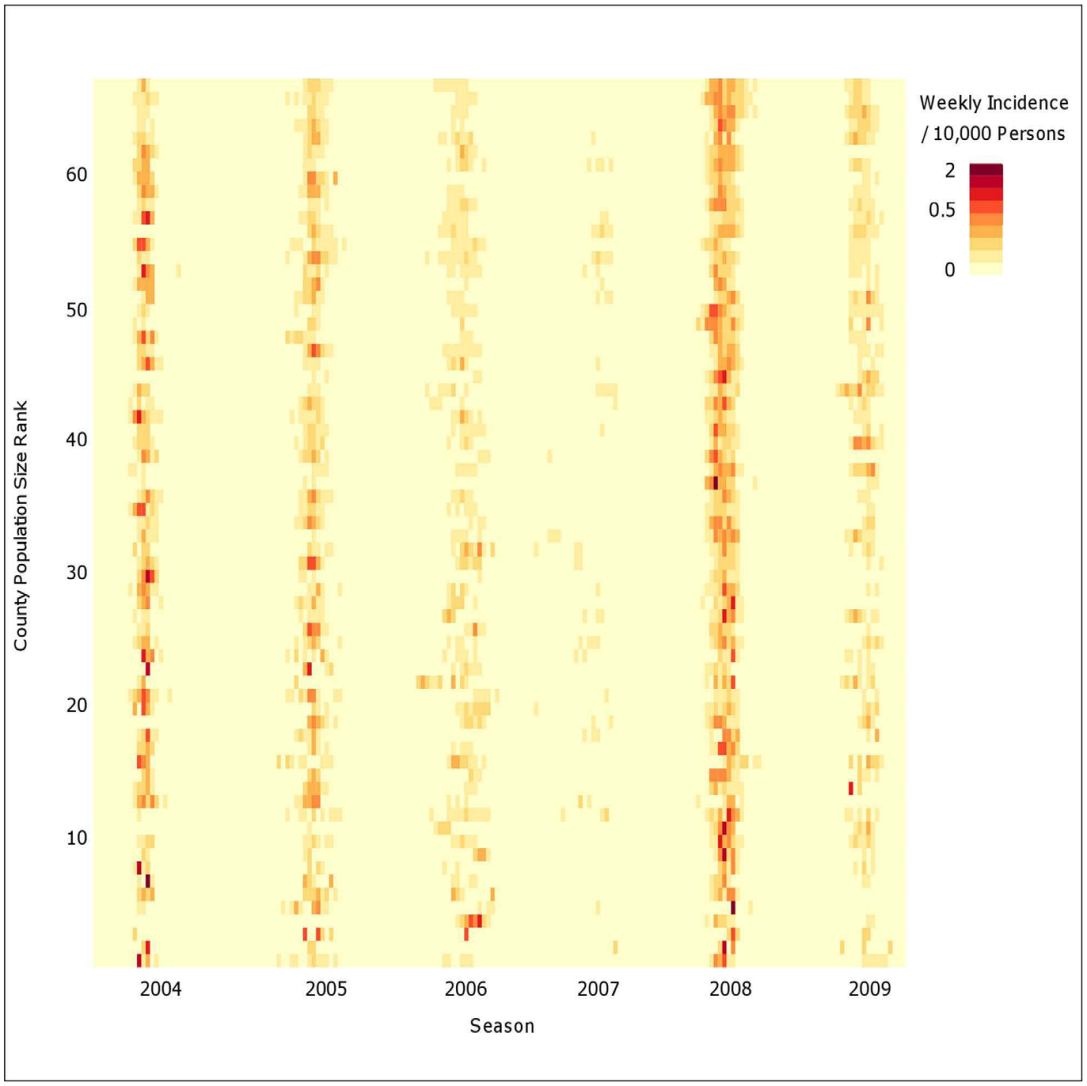
Weekly case incidence for 67 counties by population size. Intensity image displace weekly case incidence (per 10,000 persons) sorted by population size. The counties are arranged from largest population size (67 = Philadelphia) to the smallest population size (1 = Forest). The surveillance system defines each influenza season to begin in the 40^th^ week of the calendar year through the last week of April of the following year.

A map illustrating the features of Pennsylvania can be found in Figure 1. Weekly incidence of cases for all 67 counties is presented in Figure 2. The 2007/08 season experienced a particularly severe influenza season as noted by the darker color intensity. Of the 186 weeks of influenza data analyzed for each county, the mean number of weeks with at least one case was 76 weeks and the range was 11 weeks (Cameron) to 141 weeks (Allegheny). Additional statistics describing the differences between the large and small population counties were presented in Table 1. The partition of counties by population size was determined by a natural break in the data.

**Table 1:** County characteristics.

	All Counties (N = 67)	Large Counties (N = 30)	Small Counties (N = 37)
Mean population size	183,300	349,600	48,440
Population range	4,946–1,518,000	120,000–1,518,000	4,946–94,640
Total number of weeks*	12462	5580	6882
Proportion of total weeks with a case	41%	53%	31%
Mean number of weeks with a case for each county	76	98	58

* 186 weeks over 6 epidemic seasons (31*6).

Results of the binomial sampling demonstrated that sampling error has limited effect on the correlation of epidemic time series between counties. The mean correlation of the 1000 pair-wise binomial sampled correlations was 0.692 (95% CI: 0.658, 0.726). The mean Spearman correlation from the correlations of the 30 larger populated counties was 0.76 and 0.54 for the 37 smaller populated counties. Because the confidence interval of binomial sampled correlations excludes the mean correlation of the smaller counties, we concluded that differences in the correlation were more likely to reflect natural differences in county structure than in the sampling error of the smaller counties. As a result, we are confident in using the incidence data for all counties to further evaluate estimates of synchrony and the predictors of disease spread.

**Figure 3:**
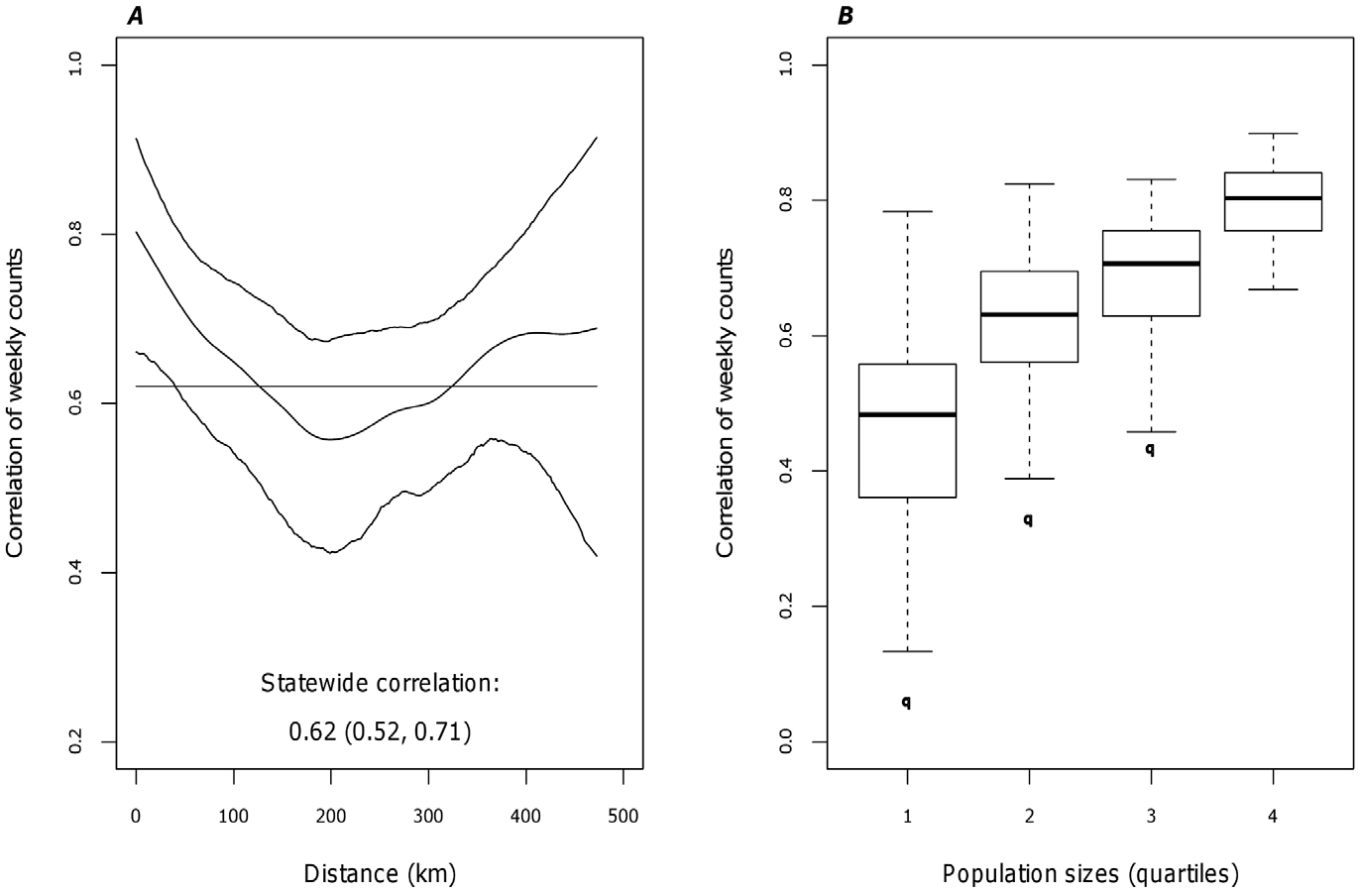
Correlation of weekly time series with distance and population size. A) Synchrony as a function of distance. The spline function (middle curve) is presented with a 95% confidence interval (outer curves). B) Synchrony as a function of population size (product of population i, j). The distribution of population was categorized by quartile. The boxplot within each quartile represent the distribution of the correlation of population between pairs of counties.

Estimation of spatial synchrony from all 67 counties used Spearman rank correlations of the epidemic time series and a distance matrix composed of county centroids. Considerable correlation existed across the entire state as the regional correlation was 62% (Figure 3A). Adjacent counties had a high mean correlation of 80%; although, synchrony declined with distance and approached the regional mean correlation at 127 km. The lower bound of the 95% confidence interval crosses the regional correlation at 36 km. Prior to this distance, the local synchrony is statistically significantly different than the state correlation. Fewer than 2% of county pairs have county centroids separated by 36 km or less, thus the correlation in epidemic time series between neighboring counties was not extensive. Seasonal analysis of synchrony as a function of distance did not note observable differences for five of the six seasons presented. The 2006–07 season had a weak correlation but similar distance trend which can be attributed to the weak epidemic season [24]. Details can be found in Figure S1. The rising, yet not significant, increase in synchrony over distance (U-shaped curve) reflected strong correlation among the larger population regions separated by several hundred kilometers.

**Figure 4:**
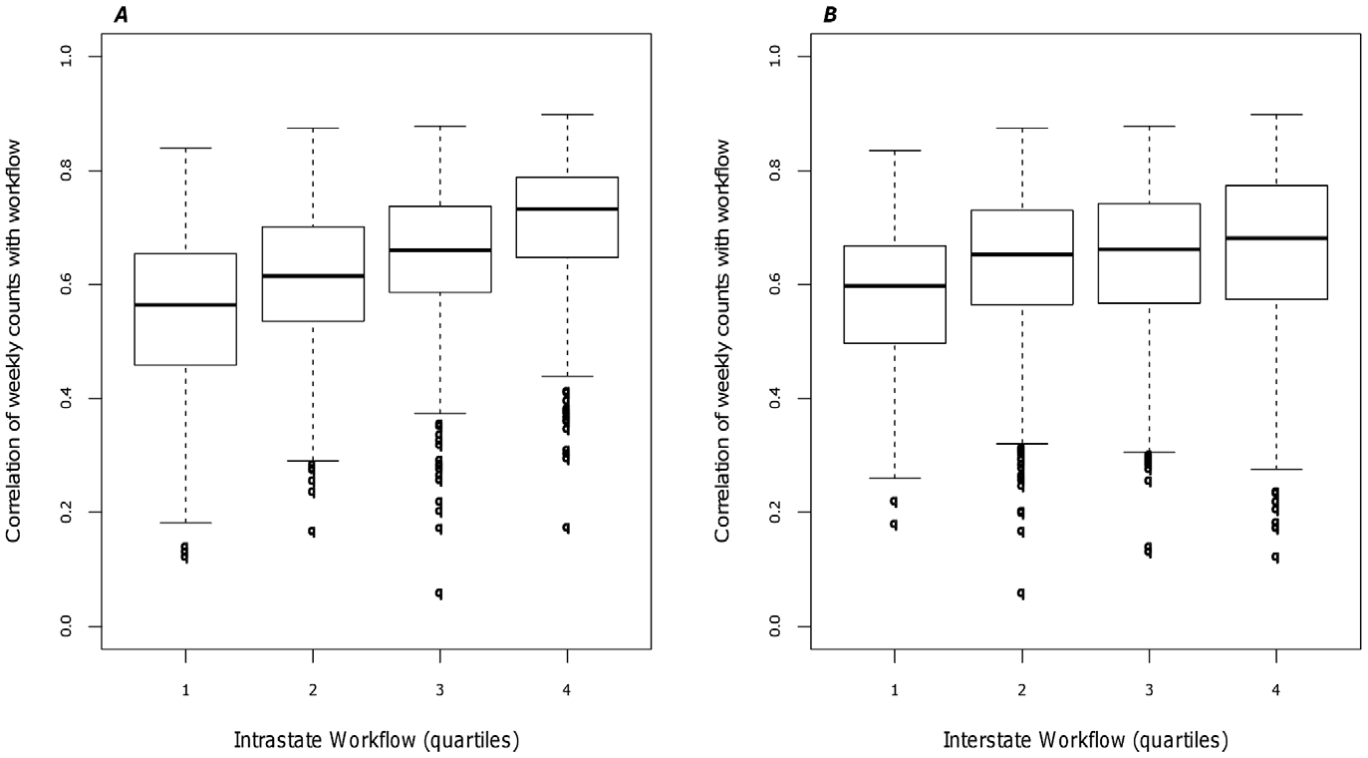
Correlation of weekly time series with human movement. A) Synchrony as a function of workflows. B) Synchrony as a function of Pennsylvania and neighboring county workflows. The distribution of workflow was categorized by quartile. The boxplot within each quartile represent the distribution of the correlation of workflow between pairs of counties.

**Figure 5:**
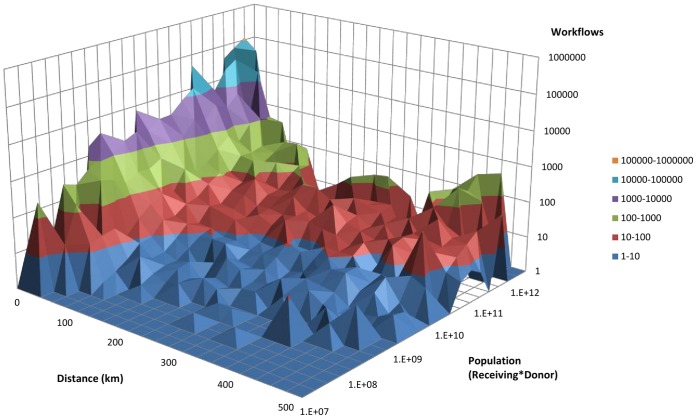
Association of workflows, population and distance. (y-axis and z-axis log10 scale). The relationship between workflows (z-axis), population size (y-axis) and distance (x-axis).

Measurement of spatial synchrony as a function of population sizes and county workflows also revealed interesting patterns. Synchrony increased as the product of the county population size increased ranging from a correlation of 0.51 in the smallest quartile to a correlation of 0.75 in the largest quartile (Figure 3B). A positive, but not significant trend existed for synchrony and county to county workflows (Figure 4A). These county-specific synchrony results were consistent with the observations of distance, population size, and workflow observed by Viboud et al. using state-specific mortality time series [6]. The inter-state workflows consisting of neighboring counties of Pennsylvania also exhibited a positive trend, though less variation between quartiles compared to the intra-state workflows (Figure 4B). Figure 5 describes the three dimensional relationship between workflows, distance, and population size.

The Mantel statistic describing the relationship between the epidemic time series and distance, population, and human movement are presented in Table 2. Only Euclidian distance was not significantly associated with influenza when evaluating all 67 Pennsylvania counties. The inter-state workflow matrix had a smaller correlation than the intra-state workflows likely implying that work-related movement of individuals from the neighboring states was not strongly associated with disease spread within Pennsylvania. Many counties in Pennsylvania did not experience work-related movements to all of the border state counties; thereby, necessarily reducing the correlations.

**Table 2:** Observed Mantel statistics.

All counties (N = 67)				
Matrix	Correlation	P-value*	Lower CI	Upper CI
Euclidian distance	−0.03	0.5528	−0.079	0.006
Workflow (Intra-state)	0.14	0.0001	0.129	0.157
Workflow (Inter-state)	0.08	0.0260	0.058	0.099
Population	0.33	0.0004	0.310	0.389
Gravity (United States)†	0.11	0.0013	0.094	0.140
Gravity (Pennsylvania – Workflows)	0.19	0.0001	0.169	0.245
Gravity (Pennsylvania - Disease)	0.63	0.0001	0.593	0.656
* Gravity(Pennsylvania - Disease) adjusting for:*				
Euclidian distance	0.63	0.001		
Workflow (Intra-state)	0.62	0.001		
Population	0.60	0.001		

Pearson correlation of the dissimilarity matrices and Spearman rank correlations of the epidemic time series for all counties (N = 67). P-values and the corresponding 95% confidence intervals (CI) are presented. Gravity Pennsylvania refers to the gravity model fitted to Pennsylvania-specific data.

† Gravity matrix generated using parameters derived from Viboud et al.

* Significance is determined at P<0.05.

**Figure 6:**
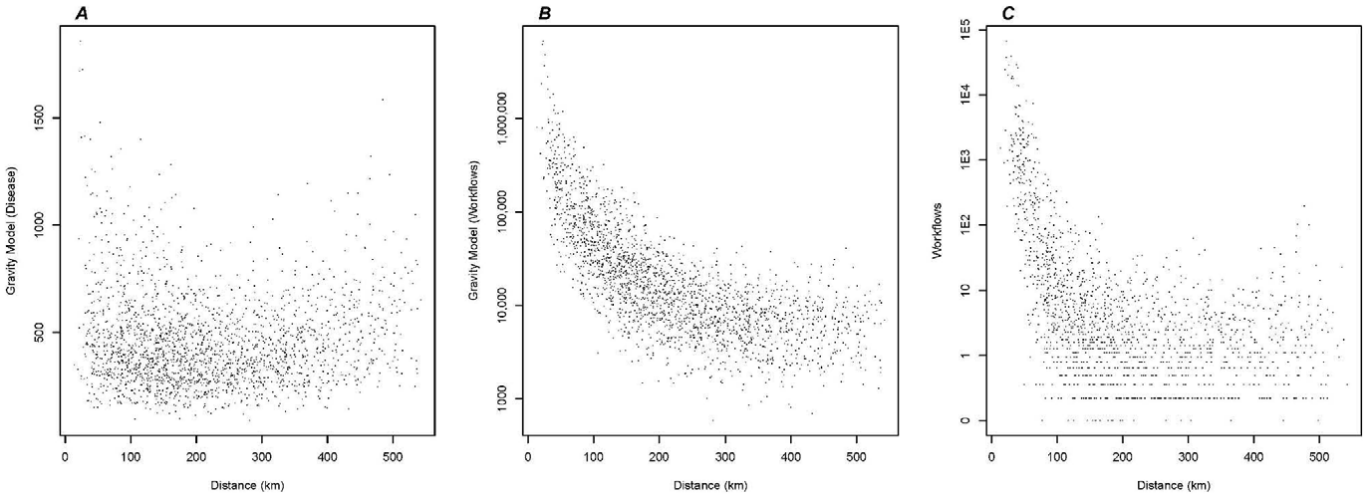
Correlation of gravity model and workflows with distance. A) Each point represents the distance between two counties as a function of the gravity model fitted to disease for the pair of counties. B) Each point represents the distance between two counties as a function of the gravity model fitted to workflows for the pair of counties (y-axis log10 scale). C) Each point represents the distance between two counties as a function of the workflows for the pair of counties (y-axis log 10 scale).

The gravity matrix fitted to Pennsylvania county disease data was the strongest predictor of influenza spread within the state. After adjusting for population size, distance, and workflows, the gravity model remained the strongest predictor of influenza spread. Similar to the 3-dimensional figure of workflows, distance, and population (Figure 5), distance as a function of the gravity model also displayed a U-shaped pattern (Figure 6A). A comparable but less pronounced trend of workflows as a function of distance was observed (Figure 6C). The gravity model fitted to Pennsylvania-specific workflows was not a strong predictor of disease spread (ρ = 0.19, p<0.001), and the trend over distance noted in the gravity model fitted to disease data did not materialize (Figure 6B). A comparison of the parameter estimates fitted by the gravity model is presented in Table 3.

**Table 3:** Parameter estimates for gravity models (disease spreads and workflows) and the corresponding 95% confidence intervals (CI).

Coefficient (exponents)		Gravity Model - Disease	Lower CI	Upper CI	Gravity Model - Workflows	Lower CI	Upper CI
τ1, τ2	Population	0.265	0.257	0.268	0.47	0.37	0.57
ρ	Distance	0.098	0.086	0.11	1.76	1.74	1.78

## Discussion

Few studies have explored synchrony of influenza epidemics and the predictors that drive influenza spread. This study further evaluated these quantities though at a finer spatial scale than previously reported. These results demonstrated evidence of spatial-temporal correlation in the incidence of influenza across counties of Pennsylvania. Significant synchrony among neighboring counties existed and a gravity model describing movement between two populations was the best predictor of influenza spread.

Comparison of these results to influenza spread in the United States could reflect differences in the mechanisms of spread at different geographic scales. Analysis of influenza incidence among the US Census regions demonstrated the importance of air travel in long-range dissemination. While adult workflows effectively captured the spread of influenza across the United States, a gravity model did better at the smaller county to county scale. Interstate commerce and other opportunities for interstate workflows may be responsible for the majority of interactions at these larger distances. Within one state, other interactions including those for errands, leisure, and school may be relatively more important. A gravity model may have captured these interactions more effectively than workflows despite observing a less pronounced pattern among workflows by distance. The small correlation between intra-state workflows and the Pennsylvania gravity model (ρ = 0.19) indicated that movement within the state was not completely dependent on workflows. This notion was further confirmed by the differences in distance as a function of gravity models fitted to disease and workflows where the movement trends did not coincide at longer distances. Thus mechanistically, work-related commuting did not account for the majority of movement at longer distances and disease synchrony within Pennsylvania, and the epidemics between counties in Pennsylvania were synchronized by non-routine travel.

Estimating the movement kernel has important implications for accurately simulating disease spread. Multiple large-scale epidemic simulations have used a gravity-like model to simulate movement patterns [25], [26]. A simulation of pandemic influenza in the United States used a power law model for commuting data at the census tract resolution and fit a distribution of travel to work distances up to 200 km reasonably well [26]. The gravity model fitted to workflows in the United States mortality analysis displayed evidence of a distance threshold whereby limited work movements occurred beyond distances of 119 km [6]. A similar distance threshold existed for the Pennsylvania gravity model fitted to workflows where work movements declined rapidly until 200 km; this further validates the movement kernel used for the simulation modeling.

A comparison of the exponents between the Pennsylvania gravity models (disease and workflows) highlighted differences in the movement kernel. As expected, for travel to work, the gravity model fitted to workflows produced larger distance and population exponents than the gravity model fitted to disease spread. The larger distance exponent reflected a rapid decline in movement which was more common with routine work commuting. The estimated distance exponent of 0.098 from the Pennsylvania gravity model fitted to disease spread was only slightly larger than 0 which indicated that movement was independent of distance which was evidenced by the U-shaped curve of distance as a function of gravity. The smaller population exponent for the gravity model fitted to disease revealed the importance of smaller populations in the movement of non-routine travel and ultimately in the spread of disease.

The gravity matrix fitted to the parameters obtained from United States gravity model did not correlate well with disease spread using Pennsylvania’s county-specific influenza data. Differences in strength of correlation between gravity matrices may be the result of local variations within Pennsylvania captured more efficiently such as the range of county size and distance. A gravity model fitted to the United States may have smoothed over these differences and concealed the variation in smaller states.

Accounting for sampling error among the smaller communities with the binomial sampling method was one approach to adjust for the inherent problems associated with passive influenza surveillance system data. However the extent of this sampling (reporting) error was not known and may not be fully accounted for in the analysis. Sampling error could have presented in the form of noncompliance in reporting, subject failure to seek testing, and severity of illness. These biases led to fewer reported cases and potentially affected the timing of the cases resulting in smaller correlations. Without data on complete reporting for any one county in Pennsylvania, it was difficult to assess the extent of the bias in the correlations. Additionally, variation in vaccination rates across the counties, particularly lower vaccination rates among rural, smaller populated counties, could have lead to an increase in the number of cases; thereby, overestimating the Mantel correlation with smaller counties [27]. County-specific vaccination rates for Pennsylvania are not known; however, vaccinations rates among the elderly (Age <65) have met the 70% Healthy People 2010 goals suggesting vaccination rates for this at-risk population were quite high [28], [29].

Determining edge effects remains a challenging task in spatial analysis. For this analysis, special concern was devoted to adult movement across state borders which necessitated the development of an intrastate workflow matrix. Incorporating a total of 302 counties from the bordering states, including Pennsylvania, resulted in minimal flow between several counties outside of Pennsylvania and those within Pennsylvania, thus, not significantly impacting the correlations with disease spread. Though, the correlation between the workflow matrices was 70%, indicating nearly a third of work-related travel occurs across the state borders and of non-neighboring states. While the correlation with this matrix was not a strong predictor of overall disease synchrony, the opportunity for border transmission still exists in the form of non-routine travel. We did not account for interstate long distance or air travel as these forms of travel are negligible for each county of the state.

Age-specific attack rates vary by influenza strain and subtype [30], [31]. Influenza B and A/H1N1 typically infect younger populations which may be more mobile within communities but are less likely to be accounted for in the workflow matrix or gravity model. Mortality analyses among the elderly have shown greater synchrony among A/H3N2 seasons than seasons dominated by A/H1N1 and B [6], [8]. Nonetheless, strain and subtype-specific analyses would further illuminate the determinants of disease spread between counties. However, small influenza B samples from each county and limited data on influenza A subtypes prevented further analysis.

This study documented the gravity-like spread of disease within the state of Pennsylvania; thus placing less emphasis on the value of administrative borders for public health prevention methods. Public health officials should target interventions to multiple counties to effectively capture the flow of residents and the spread of disease. Interventions targeted to patches of the state that display significant gravity-like spread of disease might be more efficient than statewide campaigns and provide greater public health value.

The precision gained from using county-specific disease and exposure data improved our knowledge of spatial-temporal predictors of disease spread enabling this study to delineate differences in mechanisms dependent on geographic scale. While this study incorporated workflows from neighboring states, it did not include disease data. Future studies should incorporate disease data from the neighboring states to confirm the gravity-like spread of disease across a larger administrative boundary. Through analysis of county-specific data, these results can be used to inform mathematical models of influenza spread at a narrow spatial scale.

## Supporting Information

Figure S1 **Correlation of weekly time series with distance for each influenza season.** The spline function (middle curve) is presented with a 95% confidence interval (outer curves). Each graph represents a different season.
(TIF)
