# Supplementary figures and images for: Local Variations in Spatial Synchrony of Influenza Epidemics
Source: PLoS One. 2012 Aug 16;7(8):e43528. doi: 10.1371/journal.pone.0043528 (PMC3420894; doi:10.1371/journal.pone.0043528)

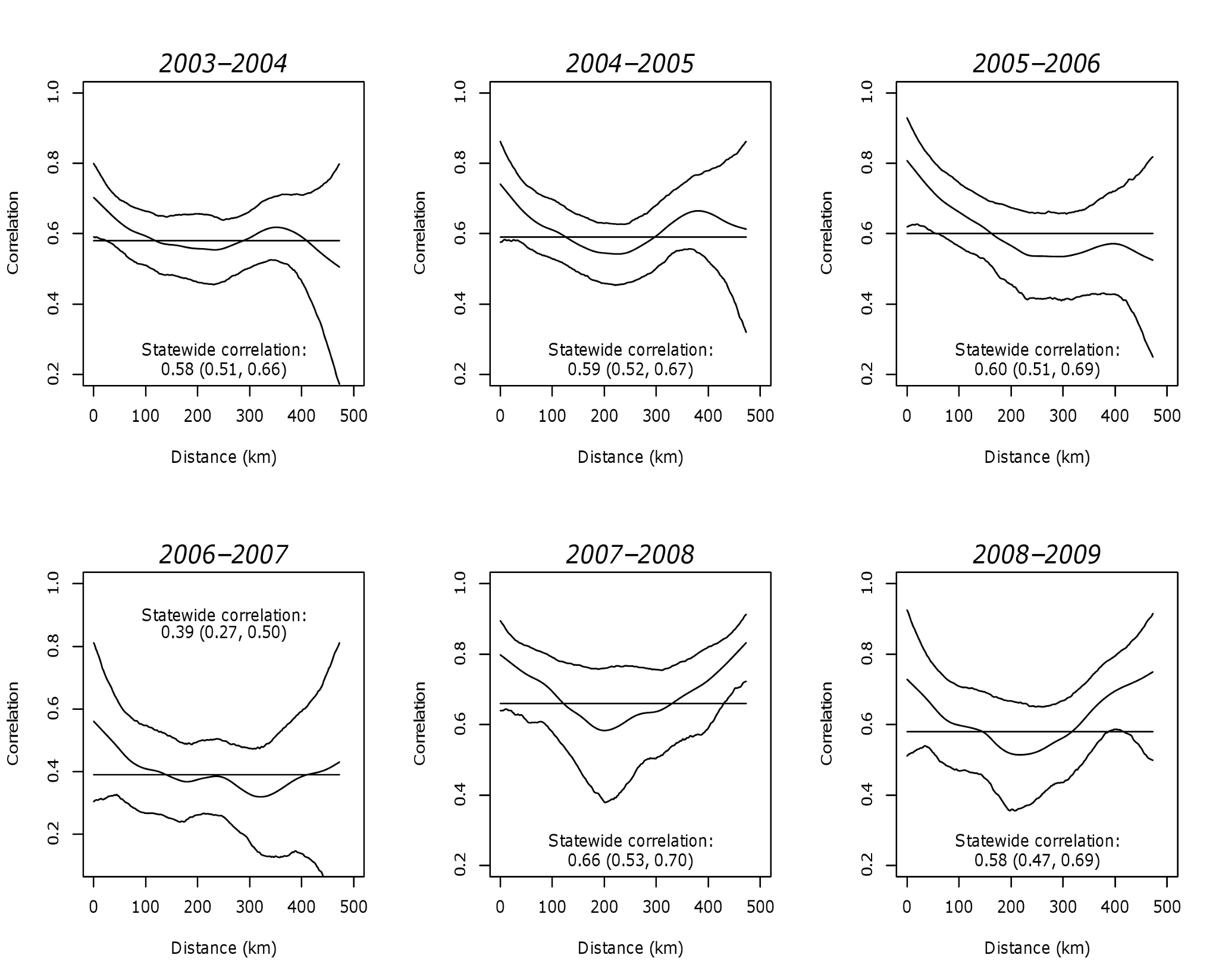

Supplement: Figure S1 — Correlation of weekly time series with distance for each influenza season. The spline function (middle curve) is presented with a 95% confidence interval (outer curves). Each graph represents a different season. (TIF) [file pone.0043528.s001.tif]
